# The prevalence and microbiological features of *Staphylococcus* species isolation from healthcare personnel in a dental clinic in Tripoli, Libya

**DOI:** 10.11604/pamj.2024.49.77.39662

**Published:** 2024-11-14

**Authors:** Hana Abdulnabi Jlaytah, Rahma Omar Ahmed, Samira Guma Amri, Ezzeddin Aghila, Mohamed Omar Ahmed

**Affiliations:** 1 Department of Biological Sciences, School of Basic Sciences, Libyan Academy of Graduate Studies, Tripoli, Libya,; 2 Faculty of Medical Technology, University of Aljfarah, Aljfarah, Libya,; 3 Faculty of Dentistry, Cairo University, Cairo, Egypt,; 4 Burns and Plastic Surgery Centre, Tripoli, Libya,; 5 Department of Microbiology and Parasitology, Faculty of Veterinary Medicine, University of Tripoli, Tripoli, Libya

**Keywords:** Dental clinics, healthcare workers, nasal colonization, methicillin-resistant *staphylococcus*, methicillin-resistant coagulase-negative *staphylococcus*, Libya

## To the editors of the Pan African Medical Journal

Methicillin-resistant *Staphylococcus aureus* (MRSA) and methicillin-resistant coagulase-negative staphylococci (MRCoNS) are increasingly reported from different healthcare settings, including dental clinics, showing variable epidemiological distribution and evolving characteristics [1]. These multidrug-resistant strains express important virulent traits, presenting public health and zoonotic [2-4]. However, data concerning dental clinical settings on these drug-resistant pathogens are insufficient and limited. The aim of the current study was to investigate the carriage and antimicrobial susceptibility patterns of *Staphylococcus* species isolated from dental healthcare workers (HCWs) in Tripoli between February and March 2022.

The HCWs were included on the basis of not suffering from any clinical conditions or being under any therapeutic treatment, including antimicrobial drugs, at least 3 months prior to sampling. Information from each participant was collected using a designated questionnaire, which included general and clinical information. A total of 274 HCWs from 38 dental healthcare settings (13 public and 25 private clinics) were volunteered. The age range was from 19 to 70 years (mean age of 31.65 years) and represented by 73% dentists (n=200/274) and 27% nurses (n=74/274). A duplicate swap sample was obtained from each volunteer using moist cotton swabs and cultured onto mannitol salt agar and Columbia-5% blood agar and incubated at 35°C for 24-48 hours. Plates were checked for typical staphylococci colonies and one typical colony from each plate was selected then overnight grown on blood agar at 35°C for 24 hours. The grown colonies were further tested by Gram stain and catalase test, and then a definite characterization was performed using a BD Phoenix automated identification and susceptibility testing system.

The level of prevalence and proportional rates was calculated by descriptive analysis using Statistical Package for Social Science (SPSS) statistics for Windows version 20 (SPSS-20, IBM Corp, Armonk, NY). The associations between categorical variables were analyzed using the Chi-square test to determine the variability between more than two groups, whereas the Z-test was used to determine the significant difference between two groups at p ≤ 0.05. A total of 32.4% (n=89/274) of HCWs were colonized with *Staphylococcus* species and 6.9% (n=19/274) were colonized with MRS strains. Although there were no significant differences between the two genders, the male group had a higher carriage rate of staphylococci and MRSA, respectively at 40% and 10%, compared to the female group's rates at 30% and 6.5%, respectively. Also, no significant difference was identified between any of the analyzed age groups, but staphylococci carriage in the first group (18 to 25 years) was higher at a rate of 34%, and the MRS carriage in the second group (26 to 44 years) was higher at a rate of 7%. Furthermore, no differences in the carriage rates of staphylococci and MRS were found between the two study groups based on the profession and type of clinic, but doctors were found to have high staphylococci and MRS carriage at 33% and 8%, respectively. Healthcare workers who worked in both the private and public sectors had high staphylococci and MRS carriage at 38% and 8%, respectively, with the HCWs group of private clinics having the highest rate of MRS at 10%. In contrast, participants who had not recently experienced infections or who had not received antibiotic treatment had higher carriage rates of staphylococci and MRS, with the respective carriage rates of 33% and 8% for both groups. However, only the carriage rate of MRS was significantly different between the studied groups of these two variables.

A total of 89 *Staphylococcus* species and subspecies were collected, represented by 22 *S. aureus* isolates and 67 coagulase negative staphylococci (CoNS) subspecies isolates distributed respectively as follows: *S. epidermidis* (n=46), *S. capitis* (n=8), *S. haemolyticus* (n=6), *S. saprophyticus* (n=3), *S. warneri* (n=2) and *S. lugdunesis* (n=1). Of these, 21.3% nineteen of the 89 isolates (19/89) had the typical MRS phenotype, represented by *S. aureus* (n=9), *S. epidermidis* (n=4), *S. haemolyticus* (n=3), and *S. saprophyticus* (n = 3) (Table 1). In the current study, 32.4% and 6.9% of the studied HCWs were colonized with *Staphylococcus* and MRSA, mostly of the CoNS group. Such rates exceeded the global prevalence estimates reported by various studies [5]. The coagulase positive staphylococci (CoPS) group was only represented by *S. aureus*, accounting for 24.7% (n=22/89) of the total collected isolates and a total HCW colonization rate of 8% (n=22/274). On the other hand, 11.2% (n=10/89) of the collected CoNS isolates were MRS (i.e., MRCoNS), representing an HCW colonization rate of 3.6% (n=10/274). Furthermore, the MRSA group showed similar MDR profiling, whereas the MRCoNS showed variability at species and antibiogram levels. This is probably attributed to the large number of species that constitute the group of CoNS within *Staphylococcus*, which plays an important role as a reservoir of species and genetic elements posing a potential risk of transferring genetic elements between different staphylococci species [6]. In the current study, six MDR CoNS expressed low susceptibility to mupirocin and were represented 4 *S. epidermidis*, a MLSB *S. epidermidis* and a MRS S. heamolyticus. These were identified using an automated microbiological system, which is a highly accurate tool to identify various healthcare-associated pathogens including the critically resistant Gram-negative rods [7-9]. The emergence of high-level mupirocin resistance is widely attributed to the repeated and/or long-term exposure of CoNS colonizing nasal mucosa to topical and indiscriminate use of mupirocin but also indicates a potential expansion of the reservoir of MDR and transferrable molecular determinants encoding mupirocin resistance such as mupA and mupB gene [10].

**Table 1:** antimicrobial susceptibility profiling of MRS strains (n=19)

Species	Origins	Source	Resistance profile	Susceptible profile
*S. aureus*	Public	Dentist	AMP, PenG, OXA, AMX, FOX, CTX, IPM	GEN, DAP, STX, TEC, VAN, CLI, ERY, LZD, MUP, NFZ, CIP, MXF, RIF, TET
*S. aureus*	Private	Nurse	AMP, penG, OXA, AMX, CTX, FOX, IPM	GEN, DAP, STX, TEC, VAN, CIT, ERY, MUP, NFZ, CIP, MXF, RIF, TET
*S. aureus*	Private	Dentist	AMP, penG, OXA, AMX, CTX, FOX, IPM	GEN, DAP, STX, TEC, VAN, CLI, ERY, LZD, MUP, NFZ, CIP, MXF, RIF, TET
*S. aureus*	Public	Dentist	AMP, penG, OXA, AMX, CTX, FOX, IPM	GEN, DAP, STX, TEC, VAN, CLI, ERY, LZD, MUP, NFZ, CIP, MXF, RIF, TET
*S. aureus*	Public	Dentist	AMP, penG, OXA, AMX; CTX, FOX, GEN, IPM	DAP, CIP, STX, TEC, VAN, CLI, ERY, LZD, MUP, NFZ, MXF, RIF, TET
*S. aureus*	Public	Dentist	AMP, penG, OXA, AMX, STX, TET, CTX, FOX, IPM	GEN, DAP, TEC, VAC, CLI, ERY, LZD, MUP, NFZ, CIP, MXF, RIF
*S. aureus*	Public	Dentist	AMP, penG, OXA, AMX, TET, CTX, FOX, GEN, IPM	DAP, STX, TEC, VAN, CLI, ERY, LZD, MUP, NFZ, CIP, MXF, RIF,
*S. aureus*	Public	Dentist	AMP, penG, OXA, AMX, CTX, FOX, IPM	GEN, DAP, STX, TEC, VAN, CLI, ERY, LZD, MUP, NFZ, CIP, MXF, RIF, TET
*S. aureus*	Public	Dentist	AMP, penG, OXA, AMX, CTX, FOX, IPM	GEN, DAP, STX, TEC, VAN, CLI, ERY, LZD, MUP, NFZ, CIP, MXF, RIF, TET
*E. cloacae*	foot	Nurse	Ceph, Cefu, fox, Ceft, Cef, Aztreonam	GEN, DAP, STX, TEC, VAN, CLI, LZD, MUP, NFZ, MXF, RIF
*S. saprophyticus*	Private	Dentist	AMP, penG, OXA, AMX, FUS, FOX, CTX, IPM	GEN, DAP, STX, TEC, VAN, CLI, ERY, LZD, MUP, NFZ, CIP, MXF, RIF, TET
*S. epidermidis*	Private	Dentist	AMP, penG, OXA, AMX, FOX, TET, CTX, IPM	GEN, DAP, STX, TEC, VAN, CLI, ERY, LZD, MUP, NFZ, CIP, MXF, RIF,
*S. epidermidis*	Private	Dentist	AMP, penG, OXA, AMX, TET, FOX, CTX, IMP	GEN, DAP, STX, TEC, VAN, CLI, ERY, LZD, MUP, NFZ, CIP, MXF, RIF
*S. haemolyticus*	Public	Dentist	AMP, penG, OXA, AMX, CTX, FOX, IPM ERY, MUP,	GEN, DAP, STX, TEC, VAN, CLI, LZD, NFZ, CIP, MXF, RIF
*S. epidermidis*	Public	Dentist	AMP, penG, OXA, AMX, CTX, FOX, IPM	GEN, DAP, STX, TEC, VAN, CLI, ERY, LZD, MUP, NFZ, CIP, MXF, RIF, TET
*S. saprophyticus*	Private	Dentist	AMP, penG, OXA, AMX, FUS, FOX, CTX, IPM	GEN, DAP, STX, TEC, VAN, CLI, ERY, LZD, MUP, NFZ, CIP, MXF, RIF, TET
*S. haemolyticus*	Private	Dentist	AMP, penG, OXA, AMX, CTX, FOX, IPM, CLI, ERY, TET	GEN, DAP, STX, TEC, VAN, MUP, LZD, NFZ, CIP, MXF, RIF
*S. epidermidis*	Private	Nurse	AMP, penG, OXA, AMX, FOX, CTX, IPM	GEN, DAP, STX, TEC, VAN, CLI, ERY, LZD, MUP, NFZ, CIP, MXF, RIF, TET
*S. saprophyticus*	Public	Dentist	AMP, penG, OXA, AMX, FUS, FOX, CTX, IPM	GEN, DAP, STX, TEC, VAN, CLI, ERY, LZD, MUP, NFZ, CIP, MXF, RIF, TET
*S. haemolyticus*	Public	Dentist	AMP, penG, OXA, AMX, CTX, FOX, IPM, ERY, TET	GEN, DAP, STX, TEC, VAC, CLI, LZD, MUP, NFZ, CIP, MXF, RIF,

IPM: imipenem, FOX: cefoxitin, CTX: cefotaxime, AMP: ampicillin, PenG: penicillin G, AMX: amoxicillin and clavulanic acid, OXA: oxacillin, ERY: erythromycin, CLI: clindamycin, TET: tetracycline, STX: trimethoprim-sulfamethoxazole, GE: gentamicin, CIP: ciprofloxacin, NFZ: nitrofurazone, MUP: mupirocin, LZD: linezolid, RIF: rifampin, DAP: daptomycin, VAN: vancomycin, TEC: teicoplanin, MXF: moxifloxacin

## Conclusion

In conclusion, this is the first study that provides important information and data of the prevalence and distribution of *Staphylococcus* species and the associated MDR phenotypes circulating in HCWs in dental clinics in Libya. Healthcare workers were significantly colonized with different MDR species of *Staphylococcus*, posing serious concern to other personnel and the general population.
